# Rationale and design of the allogeneiC human mesenchymal stem cells (hMSC) in patients with aging fRAilTy via intravenoUS delivery (CRATUS) study: A phase I/II, randomized, blinded and placebo controlled trial to evaluate the safety and potential efficacy of allogeneic human mesenchymal stem cell infusion in patients with aging frailty

**DOI:** 10.18632/oncotarget.7727

**Published:** 2016-02-25

**Authors:** Samuel Golpanian, Darcy L. DiFede, Marietsy V. Pujol, Maureen H. Lowery, Silvina Levis-Dusseau, Bradley J. Goldstein, Ivonne H. Schulman, Bangon Longsomboon, Ariel Wolf, Aisha Khan, Alan W. Heldman, Pascal J. Goldschmidt-Clermont, Joshua M. Hare

**Affiliations:** ^1^ Interdisciplinary Stem Cell Institute, University of Miami Miller School of Medicine, Miami, FL, USA; ^2^ Department of Surgery, University of Miami Miller School of Medicine, Miami, FL, USA; ^3^ Department of Medicine, University of Miami Miller School of Medicine, Miami, FL, USA; ^4^ Division of Cardiology, University of Miami Miller School of Medicine, Miami, FL, USA; ^5^ Division of Endocrinology, University of Miami Miller School of Medicine, Miami, FL, USA; ^6^ Division of Nephrology, University of Miami Miller School of Medicine, Miami, FL, USA; ^7^ Department of Otolaringology, University of Miami Miller School of Medicine, Miami, FL, USA

**Keywords:** aging, frailty, mesenchymal stem cells, allogeneic, Gerotarget

## Abstract

Frailty is a syndrome associated with reduced physiological reserves that increases an individual's vulnerability for developing increased morbidity and/or mortality. While most clinical trials have focused on exercise, nutrition, pharmacologic agents, or a multifactorial approach for the prevention and attenuation of frailty, none have studied the use of cell-based therapies. We hypothesize that the application of allogeneic human mesenchymal stem cells (allo-hMSCs) as a therapeutic agent for individuals with frailty is safe and efficacious. The CRATUS trial comprises an initial non-blinded phase I study, followed by a blinded, randomized phase I/II study (with an optional follow-up phase) that will address the safety and pre-specified beneficial effects in patients with the aging frailty syndrome. In the initial phase I protocol, allo-hMSCs will be administered in escalating doses via peripheral intravenous infusion (n=15) to patients allocated to three treatment groups: Group 1 (n=5, 20 million allo-hMSCs), Group 2 (n=5, 100 million allo-hMSCs), and Group 3 (n=5, 200 million allo-hMSCs). Subsequently, in the randomized phase, allo-hMSCs or matched placebo will be administered to patients (n=30) randomly allocated in a 1:1:1 ratio to one of two doses of MSCs versus placebo: Group A (n=10, 100 million allo-hMSCs), Group B (n=10, 200 million allo-hMSCs), and Group C (n=10, placebo). Primary and secondary objectives are, respectively, to demonstrate the safety and efficacy of allo-hMSCs administered in frail older individuals. This study will determine the safety of intravenous infusion of stem cells and compare phenotypic outcomes in patients with aging frailty.

## INTRODUCTION

Frailty is defined as a medical condition, which can be caused by multiple stressors, characterized by decreased strength, endurance, and physiologic function that increases the risk of developing dependency and/or mortality [1]. The etiology of these increased risks can be, at least partially, attributed to the critically low “reserve capacity” of various organ systems, at which point a minor disturbance can create a cascade of medically catastrophic events [2]. The syndrome has an estimated overall prevalence of 10%, with a higher prevalence found in women and in patients with chronic disease [3].

Frail individuals are major consumers of medical resources, hospitalizations, and nursing homes. Importantly, it is believed that early intervention for this medical syndrome can improve patient quality of life as well as help lower costs of care [4]. Currently, there are no specific FDA-approved treatments and thus no established pharmacologic gold standard-of-care for frail patients. Certain features of the frailty syndrome have established a potential role for adult human mesenchymal stem cells (hMSCs) to improve its symptoms. Specifically, allo-hMSCs have been shown to improve cardiac function in patients with acute myocardial infarction [5] (AMI) and chronic ischemic cardiomyopathy [6] in exert profound and sustained anti-inflammatory effects [7].

The purpose of the CRATUS study is to address several key questions related to allo-hMSC therapy and its novel use in patients with frailty. This study will test the safety of intravenous (IV) infusion of allo-hMSCs in frail subjects and will also assess the efficacy of treatment in this population at risk for morbidity and mortality.

## DISCUSSION

With a growing aging population the number of individuals in the oldest age brackets will increase at the highest rate [8]. Frailty syndrome is a common medical entity in the geriatric population and its prevalence increases with age [9, 10]. The absence of a consensus on its exact diagn ostic criteria exists together with the concomitant establishment of different models used to describe the syndrome can at least partially explain why the prevalence of frailty has varied so greatly among studies [11]—between 4% and 59.1% with a weighted average of 9.9% [3]. It has been associated with an increased risk of worse clinical outcome [12] and is known to be a major independent risk factor for falls, disability, hospitalizations, and mortality in older individuals [13]. Importantly, the clinical syndrome is dynamic, and exists as a broad spectrum. As such, the phenotype is based on an accumulation of various disabilities and comorbidities and has allowed patients to be categorized as fit, pre-frail, or frail, with varying degrees within each classification-type [14, 15]. Thus, the frailty syndrome is multifaceted in nature and derives from a complex set of interactions between physical, psychological, and social aspects of an individual.

**Figure 1:**
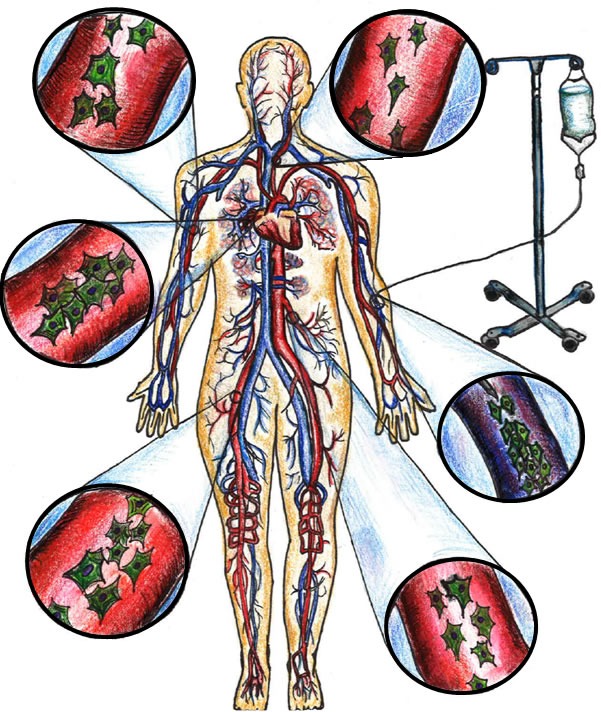
Peripheral intravenous administration of allogeneic MSCs via systemic circulation

**Figure 2:**
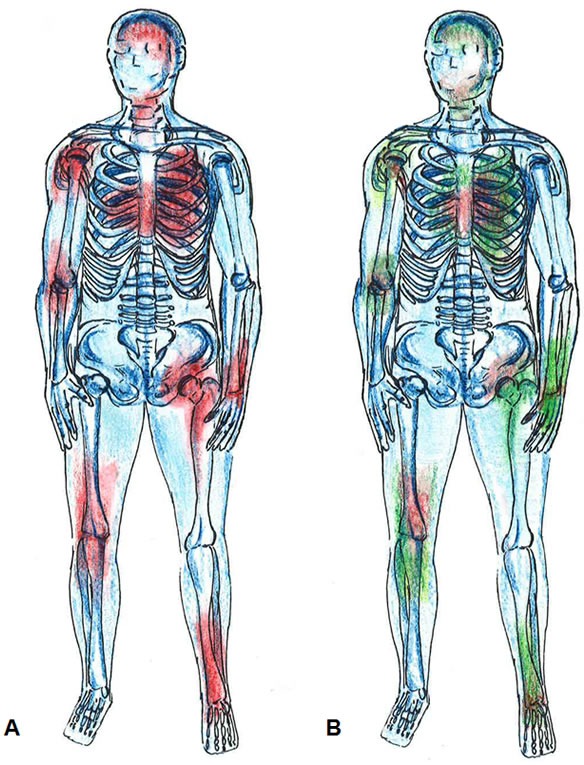
Systemic inflammation **A.** Areas in red color depict widespread inflammation. **B.** MSCs migrate to regions of injury and exert their anti-inflammatory properties (green color).

The process of aging has been characterized as having multiple hallmarks including, but not limited to, stem cell exhaustion, mitochondrial dysfunction, and altered intercellular communication related to inflammation [16]. A pathologic or accelerated process of aging can lead to the frailty syndrome [17, 18]. The decline in physiological systems is related to the abnormal and disrupted communication between them and has been biologically linked to the chronic elevation of various inflammatory cytokines, which considered one of the most notable hallmarks of frailty [19]. Indeed, several studies have indicated that elevated levels of interleukin-6 (IL-6), C-reactive protein (CRP), and white blood cell (WBC) count play a key role in systemic inflammation (Figure 2) and consequent organ dysregulation in individuals with aging frailty [20-22]. Although the cellular basis of these changes remains incompletely understood, there is an emerging field of science suggesting mitochondrial dysfunction, cellular senescence, telomere shortening, and increased levels of free radicals to be key elements in the molecular pathophysiology [23]. Moreover, endogenous stem cell production decreases with age and likely contributes to diminished organ system repair and homeostasis in frailty

Several scoring systems and indexes have been used as clinical assessment tools for frailty [9, 10, 13, 24, 25]. The Canadian Study of Health and Aging Clinical Frailty Scale [26, 27] (CSHA-CFS) is a clinical scale that measures the severity of an individual's frailty based on the physician's judgment rather than the presence or absence of specific items (i.e., grip test), which allows for a broader patient evaluation. Various investigators in numerous studies on frailty have corroborated its use in the clinical setting [28-31]. Diagnostic models and instruments have helped investigators formulate more effective treatment modalities for frail individuals. The remediation of frailty syndrome is crucial for both patient quality of life and the public health burden [32, 33]. Frailty has been described to have a continuum of “states” that frail individuals frequently transition between [34]. These changes can be bidirectional, although the switch from less severe frailty to more severe frailty is more common than the reverse. This can allow for early intervention to help delay and/or attenuate the functional decline in persons with frailty [35].

**Figure 3:**
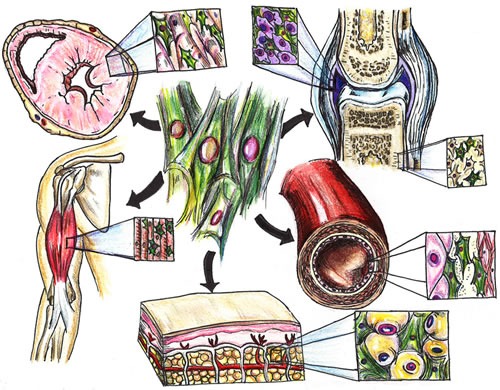
MSC effects on organ systems MSCs target various tissues throughout the body to help enhance cardiac reserve (heart), improve endothelial function (blood vessels), reduce inflammation (diffusely and in joints), and increase bone density (bone) and muscle tone (skeletal muscle) through pro-regenerative effects (i.e., paracrine signaling, mitochondrial transfer, exosomes).

A significant amount of literature [36-40] addresses the importance and validity of interventions to help prevent and attenuate frailty. Yet clinical trials have mainly focused on implementing exercise regimens [41], nutritional supplementation [42], a combination of exercise and nutrition [43], pharmacologic agents [44], or multidisciplinary interventions [45]. To date, there has been no published data on the safety and efficacy of cell-based therapy for frailty syndrome. In this regard, mesenchymal stem cell (MSC) therapy could potentially ameliorate signs and symptoms of frailty. MSCs have been particularly appealing to investigators due to its easy isolation from multiple tissues and ability to differentiate into many cell lines [46, 47]. In addition, their migratory ability and immunopriveleged state has generated much interest in its systemic application, via peripheral intravenous infusion, for a multitude of disease processes [48-51] (Figure 3). The use of allo-hMSCs, derived from young healthy donors, avoids the aging-related aberrant microenvironments of MSCs, and thus the “inflamm-aged” MSCs obtained from older individuals [6, 47, 52]. Allogeneic hMSCs not only help replenish exhausted and/or senescent native stem cells but also have demonstrated systemic anti-inflammatory properties [7] (Figure 2). Importantly, the safety of MSCs derived from healthy adult donors has been validated in numerous clinical investigations [5, 6, 53, 54]. Therefore, this therapeutic agent holds great promise as a potential, novel treatment for individuals with frailty. Furthermore, a valuable aspect of this work will be to examine and understand the mechanistic basis underlying the beneficial effects. Patients will undergo additional tests such as biomarker assessments, immune monitoring, and endothelial function assessment [55] to help elucidate mechanism of action.

## MATERIALS AND METHODS

### Study objectives

The primary objective of the study is to demonstrate the safety of allo-hMSCs in patients with frailty, and the secondary objective is to explore treatment efficacy including decrease in frailty, frequency of acute exacerbations, change in symptom-related quality of life, improved cardiovascular status, decrease in inflammatory biomarkers, endothelial function and 1-year survival. The optional follow-up phase will have the same primary and secondary objectives.

### Study design

The initial phase of this study will be non-blinded phase I study that will be performed to test the safety of the dose and volume escalation of cells administered via peripheral intravenous infusion (Figure 1). The second portion of the study will be a phase I/II blinded, randomized, placebo-controlled study that will be conducted after a full review of the safety data from the phase I by an independent Data and Safety Monitoring Board (DSMB). Approximately 15 subjects will be enrolled in the pilot phase and 30 subjects will be enrolled in the randomized phase for a total of 45 subjects.

All patients will provide written informed consent on the University of Miami Institutional Review Board-approved protocol. Upon successfully fulfilling inclusion-exclusion criteria (Tables 1 and 2) patients will receive either 20 million, 100 million, or 200 million allo-hMSCs in the lead-in phase I, and patients in the randomized phase will be randomized 1:1:1 to an active arm (100 million allo-hMSCs or 200 million allo-hMSCs) or placebo. The optional follow-up phase, in which all 15 patients of the initial pilot phase are eligible to participate, is designed to test the safety and tolerability of a second infusion of allo-hMSCs 12 to 18 months following the first infusion (100 million allo-hMSCs).

**Table 1:** Major inclusion criteria

Must provide written informed consent
Subjects age ≥60 and ≤95 years at the time of signing the informed consent form
Must show signs of frailty apart from a concomitant condition as assessed by the investigator with a frailty score of 4 to 7 using the Canadian Clinical Frailty Scale.

* Patients in the optional follow-up phase must have previously participated in the pilot phase of the trial and female subjects must have an FSH ≥ 25.8 IU/L

**Table 2:** Major exclusion criteria

Must not score ≤24 on the Mini Mental State Examination (MMSE)
Inability to perform any of the assessments required for endpoint analyses (report safety or tolerability concerns, perform pulmonary function tests (PFTs), undergo blood draw, read and respond to questionnaires
Active listing (or expected future listing) for transplant of any organ
Clinically important abnormal screening laboratory values, including but not limited to: hemoglobin <8 g/dL, white blood cell count <3000/mm3, platelets <80,000/mm3, INR >1.5 not due to a reversible cause (i.e. Coumadin), aspartate transaminase, alanine transaminase, or alkaline phosphatase >3 times the upper limit of normal, total bilirubin >1.5 mg/dL
Serious comorbid illness that, in the opinion of the investigator, may compromise the safety or compliance of the patient or preclude successful completion of the study including but not limited to: HIV, advanced liver or renal failure, class III/IV congestive heart failure, myocardial infarction, unstable angina, cardiac revascularization with the last 6 months, or severe obstructive ventilator defect
Any other condition that, in the opinion of the investigator, may compromise the safety or compliance of the patient or preclude successful completion of the study
Have known allergies to penicillin or streptomycin
Be an organ transplant recipient
Have a clinical history of malignancy within 5 years (i.e., patients with prior malignancy must be disease-free for at least 5 years) except curatively-treated basal cell carcinoma, squamous cell carcinoma, melanoma in situ, or cervical carcinoma if recurrence occurs
Have a non-pulmonary condition that limits lifespan to <1 year
Have a history of drug or alcohol abuse within the past 24 months
Be serum-positive for HIV, hepatitis BsAg or viremic hepatitis C
Be currently participating (or participiated within the previous 30 days) in an investigational therapeutic or device trial
Be a female who is pregnant, nursing, or of childbearing potential while not practicing effective contraceptive methods. Female patients must undergo a blood or urine pregnancy test at screening and within 36 hours prior to injection
Female subjects must have an FSH <25.8 IU/L
Have a hypersensitivity to dimethyl sulfoxide (DMSO)

In the randomized phase of the trial, subjects who received placebo will have the option to receive allo-hMSCs, if all endpoints are met, at which time the subjects will be administered the study drug and follow the study schedule from Day 1 to Month 12 after receiving the infusion of allo-hMSCs.

### Outcome measures for safety

The primary outcome measure for safety will include the incidence (at 1 month post-infusion) of any treatment emergent serious adverse events (TE-SAEs), defined as the composite of death, non-fatal pulmonary embolism, stroke, hospitalization for worsening dyspnea, and clinically significant laboratory test abnormalities. Continued safety and tolerability with review of clinical laboratory tests and adverse events (AEs) will be monitored at each visit for the pilot and randomized studies, through 12 months. For the optional follow-up phase, the incidence of adverse events will be described at 30-days, 6- and 12-months post second infusion.

### Outcome measures for efficacy

The secondary efficacy endpoints will be evaluated in this trial at baseline, 3-month, and 6-month follow-up visits. They will include the difference in rate of decline of frailty defined as: reduced activity (assessed via CHAMPS questionnaire), slowing of mobility (assessed via a 4 meter gait speed test and 6-minute walk test), weight loss, diminished handgrip strength (assessed via dynamometer and short physical performance battery (SPPB) assessment), and exhaustion (assessed via the multidimensional fatigue inventory (MFI) questionnaire); difference in subject quality of life assessment(s); death from any cause; exercise change in ejection fraction; and the following panel of inflammatory markers: C-reactive protein (CRP), Interleukin-6 (IL-6), D-dimer, fibrinogen, complete blood count (CBC) with differential, DNA, and tumor necrosis factor (TNF)-α. In the optional follow-up phase of the study, secondary efficacy endpoints will only be evaluated during baseline and 6-month follow-up visits. A summary of study procedure time and events can be seen in Table 3. Patient status and freedom from adverse and serious adverse events will be ascertained at 12 months by telephone contact.

**Table 3:** Time and events

Study Procedure	Screening Day -56 (±28 days)	Baseline (−4 weeks)	Day 1	*Week 2 (Day 14) (±2 days)	Month 1 (Day 30) (±2 weeks)	Month 3 (Day 90) (±2 weeks)	Month 6 (Day 180) (±2 weeks)	Month 12 (Day 365) (±2 weeks) **Phone Call Follow-up
Informed Consent	x							
Full Medical History	x							
Physical Exam	x	x	x	x	x	x	x	
12-lead (ECG)	x	x	x	x	x	x	x	
Concomitant Medications	x	x	x	x	x	x	x	x
Mini Mental State Examination (MMSE)	x						x	
Randomization		x						
Infusion Treatment (IP)			x					
Dobutamine Stress Echo Test (DSE)	x						x	
Bone Density Scan (DEXA)^8^		x					x	
FEV-1		x				x	x	
6-Minute Walk Test		x				x	x	
4-Meter Gait Speed Test^7^		x				x	x	
SPPB Assessment		x				x	x	
Dynamometer (handgrip)		x				x	x	
Smell Identification Test (UPSIT)		x				x	x	
IIEF, SQOL-F Questionnaires		x			x	x	x	
QOL Questionnaires (ICECAP, EQ-5D, SF-36, CHAMPS, MFI)		x			x	x	x	
Urinalysis	x				x	x	x	
Hemat., Chem., CBC, LFTs, INR, and other labs^1^	x		x	x	x	x	x	
HIV 1, HIV 2, Hep. B & C, and CMV	x							
Serum or Urine Pregnancy Test^2^	x		x					
Donor Screening Tests	x							
Review Adverse Events			x	x	x	x	x	x
Immune Monitoring^4^			x	x	x	x	x	
Biomarker Assessment^3^			x				x	
Optional: Brachial Ultrasound^5^		x				x		
Optional: Endothelial blood samples^6^		x				x		

Subjects have up to 4 weeks to complete all baseline visit procedures before Day 1

1 The minimal laboratory requirements for hematological, liver function and renal function include: Hematology tests- white blood cell count, platelet count, hemoglobin and hematocrit; Liver function tests- albumin, alkaline phosphatase, alanine transaminase, aspartate aminotransferase, prothrombin time, activated partial thromboplastin time, and bilirubin; Renal function tests- creatinine, creatinine clearance, blood urea nitrogen, glomerular filtration rate, sodium, potassium, chloride, carbon dioxide, and glucose; serum uric acid, pro-BNP, C-reactive protein, IL-6, fibrinogen, and D-dimer

2 A serum or urine pregnancy test will be completed within 36 hours prior to injection for females of childbearing potential

3 The following biomarkers will be analyzed: Cell-surface markers- CXCR4, C-kit, and Connexin 43; Transcriptomic/Proteome-RNA, miRNA, protein samples and telomerase, and Akt; Growth factors- Sdf-1, Notch; Function assays- cell growth rate, VEGF, and CFU assay

4 Immune monitoring for graft rejection. The following markers will be used for analysis to assess for activated T-cells based upon a CD3+CD25+ or CD3+CD69+ phenotype: CD3, CD25, CD69

5 Optional: An addition 5 lavender top tubes (EDTA) will be drawn

6 Optional brachial ultrasound to assess endothelial function

7 4-meter gait speed test will be performed twice per visit and the average of the exams will be taken

8 DEXA scan will be performed twice at each visit. The first scan will be of the hip and spine for bone density and the second will be to assess the total body composition

* Patients in the optional follow-up phase will not be assessed at Week 2

### Blinding/randomization

The second phase of the study will be a phase I/II double-blinded, randomized, placebo-controlled trial. Patients will be randomized to treatment strategy (100 million allo-hMSCs, 200 million allo-hMSCs, or placebo) in a 1:1:1 ratio via electronic randomization using the Advantage EDC system and communicated to cellular laboratory personnel who have no contact with the investigators or subjects. At the time of administration, opaque tubing will be used for study product infusion in order to maintain double-blinding.

### Donor eligibility/cell harvesting

Male or female donors between the ages of 20 to 45 will be screened at potential bone marrow (BM) donors. A maximum of 15 subjects will be evaluated by history and physical examination. A summary of the screening history and physical as well as eligibility criteria for normal donors is shown on Tables 4 and 5. Informed consent will be obtained from all potential donors. After discharge from the hospital, bone marrow donors will be contacted by the study team with a follow-up telephone call to determine the well-being and health status of the donor.

**Table 4:** Normal donor history and physical examination

History	Physical
History of malignancy Bleeding abnormalities Prior deep venous thrombosis Known cardiac or pulmonary conditions Prior blood transfusions Vaccinations Questions to identify persons at risk of infectious disease transmission Questions to identify persons at risk of transmitting hematological or immunological disease A physician will administer the National Marrow Donor Program (NMDP) Questionnaire (a donor health history screening questionnaire)	Complete physical examination Infectious disease testing including: Hepatitis B surface antigen (HBsAg) Anti-Hepatitis B core antibody (HBcAb) Anti-Hepatitis C virus antibody (HCVAb) Anti-Human Immunodeficiency Virus (HIV) antibody (HIV 1/2) Cytomegalovirus antibody (CMV) HCV/HIV Nucleic Acid test West Nile Virus Nucleic Acid test Rapid Plasma Reagin (RPR) Human T-lymphotropic Virus I/II (HTLV I/II) T. cruzi ELISA test (Chagas disease) Complete blood count (CBC) with differential Complete metabolic panel (CMP), magnesium (Mg^2+^), Calcium (Ca^2+^), and uric acid

**Table 5:** Normal donor eligibility criteria

Male and female gender
No history of malignancy
No active coagulopathy and/or hypercoagulable state
No history of cardiopulmonary conditions
Negative tests for Hepatitis B, Hepatitis C, RPR, Chagas disease, HIV 1/2, HTLV I/II, and NAT for HCV, HIV, and WNV
Hemoglobin ≥13.0 g/dL
Platelet count 140,000 to 440,000/ul
WBC 3.0 to 11.0 K/ul
No anomalies on the CBC and differential suggestive of a hematopoietic disorder
Creatinine ≤1.5 mg/dL
ALT ≤112 IU/L
Bilirubin <1.5 mg/dL
No diabetes
Systolic blood pressure (SBP) ≤170 mmHg
Diastolic blood pressure (DBP) ≤90 mmHg
No history of autoimmune disorders
Negative serum or urine pregnancy test for female donors

A total of 120 mL of BM will be obtained from each normal volunteer. BM will be aspirated from the posterior iliac crest into heparinized syringes. The mononuclear cell fraction will be isolated using a density gradient with Lymphocyte Separation Media (specific gravity 1.077). The low-density cells will be collected and washed with Plasma-LyteA containing 1% HAS. The washed cells will be sampled and viable cell numbers determined. The BM mononuclear cells will be seeded into 225 cm^2^ tissue culture flasks in alpha MEM containing 20% FBS. After 14 days of culture, passage zero (P0) cells will be harvested by trypsin treatment and expanded into 60 flasks. These flasks are incubated for a further 7 to 10 days and then the MSCs are harvested by trypsin treatment (P1 cells).

For the optional follow-up phase, allo-hMSCs will be derived from approximately 2 – 3 normal donors meeting criteria for allogeneic unrelated human bone marrow source manufactured by the University of Miami.

### Biomarker assessment

A separate 7 mL blood sample for gene expression profiling of white blood cell RNA will be obtained at the donation visit. All samples will be identified so that they can be linked to individual patient and may be stored indefinitely. Individual results will not be returned to the patient or the study physician; only aggregate data from the entire study will be disclosed.

### Immune monitoring for graft rejection

We will obtain peripheral blood samples from all patients to evaluate the presence of activated T-cells. Two heparinized tubes will be collected at different time points during the study (Day 1 prior to infusion of allo-hMSC and Month 6). Peripheral blood mononuclear cells (PMNCs) will be isolated from heparinized blood by Ficoll sedimentation and will be viably cryopreserved for planned assessments of early and late T-cell activation and B-cell subsets. Additionally, in female patients who receive allo-hMSCs, the stored baseline serum will be analyzed to evaluate the antibody responses to HLA and H-Y antigens.

### Infusion

Prior to the start of the infusion the following procedures and assessments will be conducted on the study subject:

1. Vital signs: Blood pressure, heart rate, respiratory rate, and temperature will be measured within 15 minutes prior to the initiation of infusion.
2. Oxygen saturation will be continuously monitored by pulse oximetry for at least 30 minutes prior to initiation of infusion.
3. Confirm that peripheral intravenous (IV) access is established and that the IV catheter is no smaller than 20 gauge.
4. Study personnel need to verify and document that the following pre-medications have been administered 30 minutes prior to infusion per protocol: Hydrocortisone 25-50 mg IV and Diphenhydramine 25-50 mg IV.
5. Required IV infusion materials as follows: 0.9% normal saline IV infusion bag, IV pump tubing, IV extension tubing, volumetric infusion pump, and gloves.
6. Remove 0.9% normal saline infusion bag and connect IV tubing to the volumetric infusion pump.
7. Cover the IV tubing with the blinding material provided with the infusion bag by the drug preparation technician.

During the infusion of the following procedures and assessments will be conducted on the study subject:

1. Monitor the subject continuously with pulse oximetry.
2. Hang the blinded infusion bag (investigational product should not be “piggybacked” through another line).
3. Intravenously administer the product at a rate of 2 mL/min with the study personnel present throughout the infusion process and the investigator available at the site in case an emergency arises.
4. Record the start time of the infusion bag.
5. Gently squeeze the infusion bag several times every 15 minutes to assure uniform dispersion of contents.
6. Measurement of vital signs and oxygen saturation every 15 minutes until the end of the infusion.
7. Record the total volume from the investigational product bag.

At the end of the infusion, the line will be closed and flushed with 25 mL of 0.9% normal saline into the luer lock connecter on the bottom of the bag, and then reopened to allow the infusion of the saline at a rate of 2 mL/min until completion. Vital signs will be monitored at 15 minutes, 30 minutes, 1 hour, and 2 hours post-infusion. The subject will be monitored for a minimum of 2 hours post-infusion with continuous pulse oximetry. If the oxygen saturation decreases to <90% over a continual period of 3 – 5 minutes, then supplemental oxygen may be added or increased during the 2-hr post-infusion observation period. If at the end of the 2-hour observation period a subject's oxygen saturation remains below 90%, then the subject will be provided additional oxygen to maintain a saturation of >90% at room air up to 4 hours post-infusion. After the minimum 2-hour observation period, the subject will be continuously monitored and discharged the following day if no complaints (i.e. shortness of breath or other objective signs of cardiorespiratory compromised) are experienced. Subjects not meeting criteria for discharge will be assessed by the investigator during the observation period to further determine hospitalization.

Intravenous infusion will be discontinued if the oxygen saturation does not return to >93% within 3 minutes of initiation supplemental oxygen or if the patient requires greater than 2 L/min supplemental oxygen to achieve the required saturation of >93%. Any subject whose infusion is stopped due to cardiorespiratory distress will receive no further infusions but will continue with all scheduled follow-up if such follow-up is considered safe in the opinion of the investigator.

### Dosing

During the pilot phase of the study, a total of 15 subjects will receive a single infusion of allo-hMSCs (Figure 1):

Group 1 (5 subjects) will be treated with a single administration of 2 × 10^7^ (20 million) allo-hMSCs delivered via peripheral intravenous infusion.
Group 2 (5 subjects) will be treated with a single administration of 1 × 10^8^ (100 million) allo-hMSCs delivered via peripheral intravenous infusion.
Group 3 (5 subjects) will be treated with a single administration of 2 × 10^8^ (200 million) allo-hMSCs delivered via peripheral intravenous infusion.

In the randomized phase, a total of 30 subjects will be randomized in a 1:1:1 ratio to one of two treatment strategies or placebo following completion of the pilot phase:

Group A will consist of 10 subjects that will receive 1 × 10^8^ (100 million) allo-hMSCs delivered via peripheral intravenous infusion.
Group B will consist of 10 subjects that will receive 2 × 10^8^ (200 million) allo-hMSCs delivered via pheripheral intravenous infusion.
Group C will consist of 10 subjects that will receive placebo via peripheral intravenous infusion.
After subjects complete their Month 12 follow-up phone call visit in the pilot phase, all 15 subjects will then have the option of receiving a second single infusion of allo-hMSCs. Subjects will be treated with a single administration of 1 × 10^8^ (100 million) allo-hMSCs delivered via peripheral intravenous infusion.

### Statistical analysis

All statistical tests will be performed at an α =0.05 level of significance, using two-sided tests. Because this is a phase I/II study with exploratory efficacy outcomes, no adjustments will be made for multiple analyses, as previously discussed [56]. Continuous variables will be presented by descriptive statistics. Categorical variables will be presented by counts. Two-sided 95% confidence intervals will be calculated and presented where appropriate.

Analysis of AEs will include tabulation by frequency, severity, organ system affected, and relationship to study exposure. Lung function data will be summarized descriptively. Patient reported outcome data will be summarized according to the guidelines of each questionnaire. Comparison of AE and SAE rates will be evaluated between cell groups and placebo using Fisher Exact Test.

### Safety and monitoring

Interim analyses will be conducted at times coincident with regularly scheduled meetings of the DSMB at approximately six-month intervals. The DSMB Chair will be notified each time and SAE occurs. After all patients in in the pilot phase have been followed for 30 days, an independent DSMB will review all available data to make an independent recommendation to either keep the specified randomized dose 1:1:1 or to recommend a dose modification for the randomized placebo study.

## CONCLUSION

In conclusion, the CRATUS study is designed to demonstrate the safety of allo-hMSCs administered in patients with frailty. The results of this study will also provide novel information about the efficacy and mechanism of action of cell therapy in this important elderly population with frailty syndrome.
